# Trends and Economic Implications of Operative Management for Pediatric Forearm Fractures: A Retrospective Study

**DOI:** 10.7759/cureus.78174

**Published:** 2025-01-29

**Authors:** Ryan Lebens, Gregory I Sacks, Nathan Lebens, Rigel P Hall, James Barsi

**Affiliations:** 1 Orthopaedic Surgery, Stony Brook University, Stony Brook, USA; 2 Neuroscience, University of California, Los Angeles, USA; 3 Orthopaedic Surgery, Creighton University School of Medicine, Phoenix, USA

**Keywords:** fiscal analysis, forearm fractures, fracture management, non-operative treatment, pediatric forearm fractures

## Abstract

Introduction: Pediatric forearm fractures are traditionally managed conservatively with satisfactory results. However, operative management is trending upward without definitive evidence of superior outcomes. This investigation aims to evaluate trends in the operative management of acute pediatric forearm fractures globally and to estimate the cost of treatment to ascertain management preferences and guidance.

Methods: A retrospective cohort analysis of the TriNetX database (TriNetX, Cambridge, MA, USA) was performed by querying all pediatric patients (ages one to 21) who underwent treatment for radial and ulnar shaft forearm fractures between 2014 and 2024 (n = 30,449). The operatively managed cohort included the percentage of patients who received open treatment (current procedural terminology (CPT): 25575 and/or 25574) each year while the conservatively managed cohort featured patients who received closed treatment (CPT: 25560 and/or 25565) each year. Incidence data for 2024, up to September 4th, was extracted but not included in the trend analysis due to its incomplete representation of the full year. The annual Medicare-designated relative value units and reimbursement conversion factors were utilized to estimate cost. This approach, i.e., utilizing reimbursement rates as proxies, enables a standardized comparison of the financial implications of operative versus conservative treatments over the study period.

Results: From 2014 to 2023, the percentage of fractures managed operatively increased from 13.6% to 17.88%, with a total increase of 4.28% (p-value = 0.001). The reimbursement rate of non-operative treatment increased by 8.56% in treatments without manipulation (CPT: 25560) and a 1.58% increase in treatments with manipulation (CPT: 25565). The reimbursement of operative procedures was essentially unchanged from 2014 to 2024, with an increase ranging from 0.02% (CPT: 25575) to 0.56% (CPT: 25574). From 2014 to 2024, operative treatment had a reimbursement rate ranging from $999.01 to $1349.64, while the rate for conservative treatment ranged from $427.69 to $780.72.

Conclusion: The previously established higher reimbursement rate for operative treatment is confirmed by this study. From 2014 to 2023, the higher cost of operative treatment, represented by higher reimbursement rates, and a statistically significant increase in the percentage of patients who underwent operative treatment for pediatric forearm fractures drove up the cost of treatment. The sequelae of an operative preference are largely unknown but include increased treatment costs without evidence-based claims of superior outcomes.

## Introduction

The most common orthopedic injuries in pediatric patients are fractures of the forearm, with an annual incidence of 738.1/100,000 individuals per year, which comprises 74% of pediatric upper extremity fractures [1,2]. Fractures of both the ulna and radius, or both-bone forearm fractures (BBFFs), specifically account for roughly 30% to 50.1% of pediatric fractures of the upper extremity [3,4]. These injuries typically occur when a child experiences a fall on an outstretched hand with simultaneous application of rotational force [3,4]. Pediatric forearm fractures are classically managed through closed reduction and long-arm casting, with surgical fixation required only once conservative management has failed [5]. Such non-operative management can be utilized for BBFFs, regardless of whether the radius and ulna have been completely fractured [2,6].

Once indicated, there are several different methods available for surgical management. One of the most common techniques involves closed reduction followed by fixation by placing elastic titanium intramedullary nails. These nails allow for minimal invasiveness and can be easily removed following the completion of bony union [3]. Open reduction followed by plate fixation represents an alternative surgical approach. While plate fixation requires a larger degree of soft-tissue dissection, it has been associated with decreased complication rates relative to intramedullary nailing [7]. Surgeons may also choose to combine these two methods to decrease the risk of nonunion. This hybrid method has also been shown to decrease operative time relative to plate fixation alone [3,6-8].

As previously stated, historical standards of care have called for conservative management of pediatric forearm fractures due to the higher remodeling capabilities [9]. Despite this, recent literature has demonstrated an upward trend of operative treatment for forearm fractures [10-12]. Most current studies are steadfast that conservative treatment of pediatric forearm fractures should remain the standard of care. Of note, a study by Eismann et al. concluded that most studies presented at the Pediatric Orthopaedic Society and American Academy of Orthopaedic Surgeons conferences advocated against the aggressive treatment of forearm fractures [13]. While less common, a few studies have asserted that operative and conservative treatment yield similarly efficacious clinical results [14,15]. The past inconsistencies seen between clinical research and clinical treatment warrant further investigation. Namely, existing literature does not adequately address trends before and after the COVID-19 pandemic, nor does it thoroughly analyze the financial implications of operative versus non-operative treatments. This gap is vital, as healthcare institutions faced significant disruptions during the pandemic, which undoubtedly affected the management of forearm fractures. Furthermore, changes in children's activity levels during this period may have influenced the incidence and nature of these injuries. By comparing treatment trends before and after the pandemic, this study helps to illuminate how such global events can alter clinical practices and their economic consequences, providing vital insights into healthcare delivery during times of crisis.

Here, we conducted a retrospective cohort study including patients aged 21 and under with diaphyseal fractures of both the radius and ulnar shafts between 2014 and 2024. The data was extracted using the national health network database. Trend analysis was then done between 2014 and 2023; data from 2024 was not used for trend analysis due to incomplete representation of the full year. This article was previously presented as a meeting abstract at the 2024 Eastern Orthopaedic Association Annual Conference on October 16th, 2024.

## Materials and methods

Data source

TriNetX (TriNetX LLC, Cambridge, MA, USA) is a globally recognized federated health research network that facilitates access to electronic medical records (EMRs) from over 170 prominent healthcare organizations (HCOs) worldwide, encompassing data from more than 400 million patients. TriNetX aggregates real-time electronic health data, integrating a comprehensive range of information, including diagnoses, procedures, medications, laboratory values, genomic data, and billing codes from the International Classification of Diseases, Tenth Revision (ICD-10), and current procedural terminology (CPT). The present analysis was conducted on a dataset sourced from the US collaborative network within TriNetX, comprising 69 HCOs and covering a patient population exceeding 114 million individuals. These HCOs contribute data voluntarily, allowing their de-identified information to be queried for research purposes within the TriNetX platform without receiving any financial compensation.

For this study, data from 59 HCOs, including hospitals, primary care facilities, and specialist practices within the TriNetX network, were analyzed. The HCOs were selected by the TriNetX platform based on their participation in the US collaborative network and their active reporting status at the specific time of data extraction for the present study. As the database only contains de-identified patient information, institutional review board (IRB) approval was not required. All de-identified data accessed via the TriNetX platform complies with the Health Insurance Portability and Accountability Act (HIPAA) privacy regulations. Statistics were done within the TriNetX platform. Further information regarding the TriNetX platform can be found on its website: https://trinetx.com.

Data collection and patient cohort

The TriNetX database was retrospectively queried on September 4, 2024. Patient cohorts and outcome measures were defined using a combination of ICD-10 and CPT codes. The study focused on patients aged 21 or younger who were diagnosed and treated for radial and ulnar shaft fractures between January 1, 2014, and September 4, 2024. Age criterion was strategically chosen to encompass the entire population that might experience pediatric-specific health issues. Including patients up to age 21 allows for a comprehensive analysis of fracture treatment across the full spectrum of patients seen in pediatric care settings, which is the primary goal of the present study. 

Patients were identified as those who underwent treatment for radial and ulnar shaft fractures. Patients with any of the following were included in the study: closed treatment of radial and ulnar shaft fractures; without manipulation (CPT: 25560), closed treatment of radial and ulnar shaft fractures; with manipulation (CPT: 25565), or open treatment of radial and ulnar shaft fractures, or with internal fixation (CPT: 25575 and 25574). To ensure consistency and minimize variability in fracture types, only patients who received treatment for both radial and ulnar shaft fractures were included in the analysis. Lastly, two-tailed Z-tests were run on demographic factors and outcome differences between surgical and non-surgical cohorts. 

Incidence analysis

The primary objective of the outcome analysis is to evaluate the annual incidence rates of surgical versus non-surgical treatment of radial and ulnar shaft fractures from 2014 to 2024. The surgical treatment cohort consisted of patients who underwent open treatment of radial and ulnar shaft fractures with internal fixation, as defined by CPT codes 25574 and 25575. The non-surgical cohort included patients who received closed treatment of radial and ulnar fractures, with or without manipulation, as indicated by CPT codes 25565 and 25560. Patients who had undergone surgical treatment were excluded from the non-surgical cohort to ensure a clear distinction between the two treatment modalities. The analysis was conducted on an annual basis, starting from January 1, 2014, and extending to December 31 of each year, up to the data extraction date of September 4, 2024. The incidence of surgical and non-surgical treatments was calculated separately for each year. All patient-related data, including incidence rates, were extracted and calculated using the TriNetX platform’s advanced analytical tools. Incidence data for 2024, covering up to September 4th, was extracted but not included in the trend analysis due to its incomplete representation of the full year. After completion of the data extraction, a two-tailed Z test was run on incidence rates from 2014 to 2023 to determine the statistical significance of the trend. 

Financial cost analysis

Simultaneously, a financial cost analysis was performed using fiscal data publicly available through the Centers for Medicare & Medicaid Services (CMS) and the American Medical Association (AMA) website [16,17]. The cost of treatment was calculated annually for each CPT code utilized in the outcome analysis, starting from 2014. The analysis considered various components of the relative value unit (RVU) system, including work RVUs, non-facility RVUs, facility RVUs, and malpractice RVUs. The total RVU for each year was computed as the sum of these individual RVUs and was subsequently multiplied by the annual conversion factor to estimate the reimbursement rates for each corresponding CPT procedure. The annual reimbursement rates for each procedure were calculated using this formula, enabling a comprehensive analysis of trends and changes in the cost of treatment over the 10 years.

## Results

The query population consisted of 31,383 patients aged 21 and under who were treated with a radius and ulnar shaft fracture from January 2014 to September 2024, extracted from the TriNetX database. 5,469 of these patients received surgical treatment of their radius and ulnar shaft fracture with internal fixation (Open Treatment CPT Codes: 25574 and 25575). 25,914 of the queried patients received non-surgical treatment only for their radius and ulnar fracture (Closed Treatment CPT Codes: 25560 and 25565). 

The average age of patients who received surgical treatment was 12.46 years of age at index, with the average age of patients receiving non-surgical treatment being 7.5 years of age. Racially, most of the patients identified as white in both cohorts. Lastly, both cohorts had a significantly higher percentage of males compared to females (Table 1).

**Table 1:** Demographics of patients treated surgically and non-surgically The p-values and corresponding values of Z for the two-tailed Z-tests are also shown.

Demographic	Surgical treatment (n = 5,469)	Non-surgical treatment (n = 25,914)	p-value (value of Z)
Sex			
Male	3,923 (71.73%)	16,237 (62.66%)	<0.01 (12.72)
Female	1,473 (26.93%)	9,291 (35.85%)	<0.01 (-12.72)
Unknown	153 (2.79%)	386 (1.49%)	<0.01 (6.77)
Age at index (years)	12.6 +/- 4.2	7.5 +/- 3.67	<0.01 (1.19)
Race			
White	3,957 (72.35%)	18,016 (69.52%)	<0.01 (4.15)
Black	407 (7.46%)	2,411 (9.3%)	<0.01 (-4.38)
Asian	167 (3.05%)	813 (3.14%)	0.75 (-0.32)
American Indian or Alaska Native	31 (0.57%)	131 (0.51%)	0.57 (0.58)
Native Hawaiian or Pacific Islander	32 (0.59%)	103 (0.40%)	0.05 (1.93)
Unknown	499 (9.12%)	2,771 (10.69%)	<0.01 (-3.45)
Other	375 (6.86%)	1,669 (6.44%)	0.27 (1.13)

**Figure 1:**
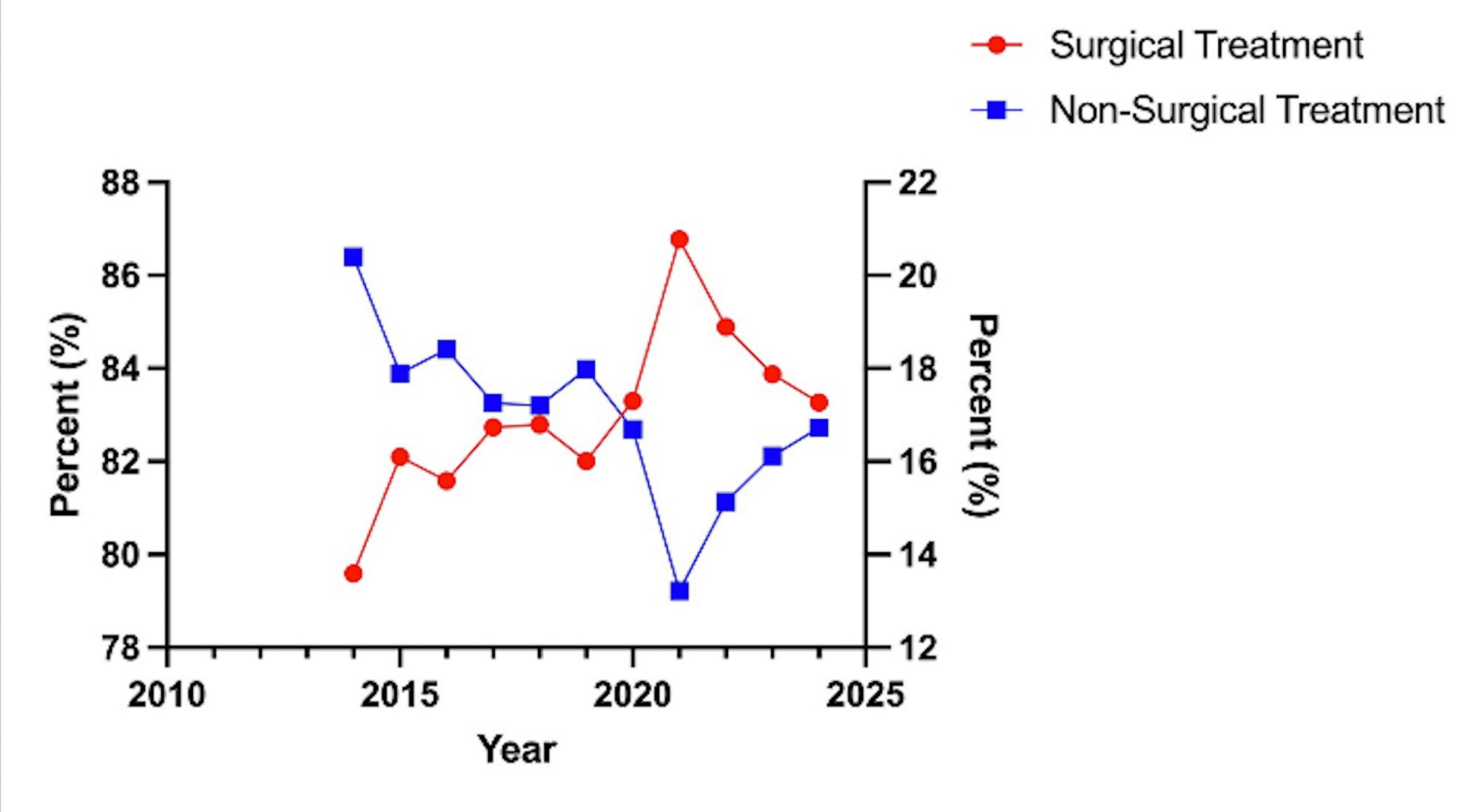
Incidence rate (%) of surgical vs. non-surgical treatment from 2014 to 2024

While data from 2024 was obtained in the results, it was not considered in the discussion of treatment trends due to not having data from the entire calendar year. From 2014 to 2023, the percentage of fractures managed surgically rose in incidence from 13.6% to 17.87%. A two-tailed Z-test comparing these incidence rates yielded a statistically significant difference, with a p-value of 0.001. 1836 (86.4%) of patients were treated with non-surgical management in 2014, with 3115 (82.12%) managed non-surgically in 2023. Peak incidence for surgical management was seen in 2021, with 731 (20.77%) patients receiving surgical management. Two-tailed Z-tests from 2014 to 2021 revealed a statistically significant difference, with a p-value less than 0.001. Non-surgical treatment had a minimum incidence rate in 2021, with 2787 (79.11%) receiving non-surgical treatment (Table 2). Overall, the percentage of patients treated surgically increased in incidence every year from 2014 to 2021. 2021 to 2022 saw a 1.89% decrease in surgical management incidence, and 2022 to 2023 saw a 1.02% decrease in surgical management incidence. Despite this, the increase in surgically managed forearm fractures from 2014 to 2023 was 4.27% (p-value = 0.001, Table 2, Figure 1).

**Table 2:** Cost of treatment trends by year for surgical treatment (CPT: 25574 and 25575) and non-surgical treatment (CPT: 25565 and 2556) The p-values and the corresponding value of Z for the two-tailed Z-test are shown to assess for differences in operative treatment rates between consecutive years.

Year	No. of patients	Surgical treatment (patients with outcome)	Surgical Treatment (%)	p-value (value of z)	Non-surgical treatment (patients with outcome)	Non-surgical treatment (%)
2014	2125	289	13.60		1836	86.4
2015	2341	377	16.10	0.02 (-2.35)	1964	83.90
2016	2817	439	15.58	0.61 (0.51)	2378	84.42
2017	3017	505	16.74	0.23 (-1.20)	2512	83.26
2018	2918	490	16.79	0.95 (-0.06)	2428	83.21
2019	3291	527	16.01	0.41(0.83)	2764	83.99
2020	2843	492	17.31	0.17 (-1.36)	2351	82.69
2021	3518	731	20.78	<0.01 (-3.50)	2787	79.22
2022	3361	635	18.89	0.05 (1.96)	2727	81.14
2023	3793	678	17.88	0.27 (1.11)	3115	82.12
2024	2252	389	17.27	0.60 (0.59)	1863	82.73

**Figure 2:**
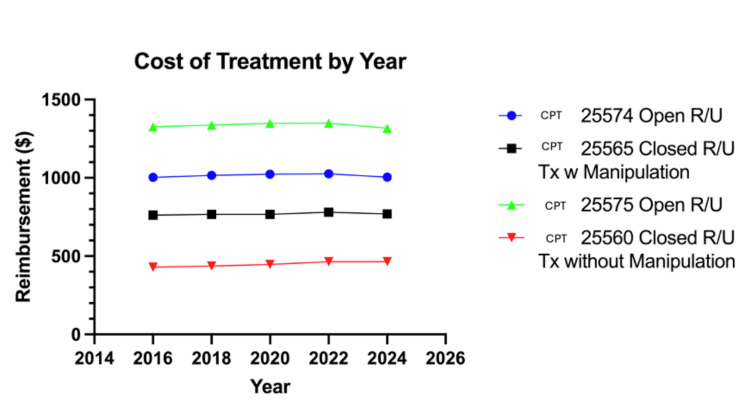
Cost of treatment (reimbursement rates in $) from 2014 to 2024 for the four examined CPT codes RVU: Relative value unit, CPT: Current procedural terminology, R/U: Radius/ulna, Tx: Treatment

From 2014 to 2024, the reimbursement rate of non-operative treatment had an increase of 8.56% in treatments without manipulation (CPT 25560) and a 1.58% increase in treatments with manipulation (CPT 25565). The reimbursement of operative procedures was essentially unchanged from 2014 to 2024, with an increase ranging from 0.02% (CPT 25575) to 0.56% (CPT 25574). For CPT 25575, there was a decrease in the reimbursement rate from $1325.48 to $1316.64 from 2016 to 2024, with the peak reimbursement rate being $1349.64 in 2022. For CPT 25574, there was an increase in reimbursement from $1003.6 to $1004.59, with a peak reimbursement being $1026.07 in 2022. Non-surgical management saw a similarly insignificant variation. For CPT 25560, reimbursement saw an increase in reimbursement from $429.65 in 2016 to $464.31 in 2024. Lastly, CPT 25565 saw an increase in reimbursement from $762.27 in 2016 to $769.16 in 2024, with a peak reimbursement of $780.72 in 2022. Overall, from 2014 to 2024, operative treatment had a cost of treatment ranging from $999.01 to $1349.94, with the cost of non-operative treatment ranging from $427.69 to $780.72 (Table 3, Figure 2).

**Table 3:** Work RVU, non-facility RVU, facility RVU, malpractice RVU, conversion factor, and total reimbursement by year for each respective CPT code used RVU: Relative value unit, CPT: Current procedural terminology

CPT code	Year	Work RVU	Non-facility RVU	Facility RVU	Malpractice RVU	Total RVU	Conversion factor	Reimbursement
25560	2014	2.59	4.82	4.07	0.46	11.94	35.82	427.69
25560	2016	2.59	4.86	4.11	0.44	12	35.80	429.65
25560	2018	2.59	4.96	4.17	0.43	12.15	35.99	437.39
25560	2020	2.59	5.08	4.24	0.48	12.39	36.09	447.15
25560	2022	2.59	5.64	4.69	0.51	13.43	34.6062	464.76
25560	2024	2.59	6.03	5.05	0.51	14.18	32.74	464.31
25574	2014	8.8	8.71	8.75	1.63	27.89	35.82	999.01
25574	2016	8.8	8.75	8.75	1.73	28.03	35.8	1003.6
25574	2018	8.8	8.85	8.85	1.71	28.21	35.99	1015.55
25574	2020	8.8	8.93	8.93	1.69	28.35	36.09	1023.14
25574	2022	8.8	9.55	9.55	1.75	29.65	34.60	1026.07
25574	2024	8.8	10.06	10.06	1.76	30.68	32.74	1004.59
25565	2014	5.85	7.80	6.44	1.05	21.14	35.82	757.23
25565	2016	5.85	7.88	6.49	1.07	21.29	35.80	762.27
25565	2018	5.85	7.93	6.49	1.02	21.29	35.99	766.43
25565	2020	5.85	7.87	6.4	1.13	21.25	36.09	766.90
25565	2022	5.85	8.57	6.94	1.2	22.56	34.60	780.72
25565	2024	5.85	9.08	7.35	1.21	23.49	32.74	769.16
25575	2014	12.29	11.11	11.11	2.24	36.75	35.82	1316.39
25575	2016	12.29	11.15	11.15	2.43	37.02	35.80	1325.48
25575	2018	12.29	11.23	11.23	2.39	37.14	35.99	1337.02
25575	2020	12.29	11.36	11.36	2.35	37.36	36.0	1348.31
25575	2022	12.29	12.12	12.12	2.47	39	34.61	1349.64
25575	2024	12.29	12.73	12.73	2.46	40.21	32.74	1316.64

## Discussion

Using the TriNetX global research database, the present study demonstrates an increase in the incidence of surgically treated radial and ulnar shaft fractures. This trend is consistent with findings in the literature suggesting that operative treatment has increased in frequency over the last two decades. Namely, Cruz et al. retrospectively found an increase in open treatment incidence rate from 59.3% in 2000 to 70% in 2012 [10]. Another study by Flynn et al. found a seven-fold increase in operative treatment over 11 years at a level 1 pediatric trauma center [11]. Specifically, from 2014 to 2023, the present study reports a 4.28% (two-tailed Z-test p-value = 0.001) increase in operative treatment and a consequent 4.27% decrease in the percentage of patients treated non-operatively. Importantly, while included in Table 2, the present study excludes the year 2024 in the trend analysis, as complete data for the entire calendar year was unavailable at the time of data extraction. 

The implications of operative treatment incidence are of particular significance when considered alongside the reimbursement rates for the corresponding years. While the present study found essentially unchanged reimbursement rates from 2014 to 2024 for both operative and non-operative treatment, the cost associated with surgery continued to be significantly higher. Namely, closed treatment without manipulation had a peak reimbursement rate of $464.76 in 2022 and saw only an increase of $36.62. Closed treatment with manipulation proved to be slightly more costly, with a peak price of $780.72, with a total increase being only $11.93 from 2014 to 2024. In conjunction with these results, surgical treatment had a range of $990.01 to $1349.64 from 2014 to 2024, demonstrating a significantly higher minimum reimbursement, even when compared to the maximum reimbursement of closed treatment. Overall, the sustained higher cost of surgical management, coupled with the respective increase in surgical treatment incidence and decrease in non-surgical treatment, indicates an increase in the cost of treatment for forearm fractures from 2014 to 2024. This finding is of great importance given the high incidence of forearm fractures in pediatric populations [1,2]. 

Given these findings, it’s crucial to evaluate the clinical viability of operative versus non-operative treatment in ongoing, past, and future research. Overall, the current literature appears to have an unclear stance on whether operative or non-operative treatment is more clinically efficacious, with various studies supporting both modalities. Firstly, there are undoubtedly studies showing an increased complication rate for forearm fractures treated non-surgically, with Sinikumpu et al. finding in a 2013 study that loss of reduction requiring additional reduction or surgery was twice as high in diaphyseal forearm fractures after non-invasive versus invasive treatment [18]. In another study from 2022, Aladraj et al. found similar success between surgically and conservatively managed distal radius fractures. However, they did observe a higher rate of needing subsequent surgery or reduction in the conservative group [19].

Despite various studies demonstrating a higher complication rate in conservative treatment, there exists equally convincing support for non-operative treatment. Specifically, a study by Sinikumpu et al. assessing the clinical and radiographic outcomes of forearm shaft fractures in children aged nine to 14 years found that the long-term outcomes of non-operatively treated fractures were excellent [20]. Additionally, Liu et al. found in a 2021 study no difference in pediatric forearm fracture angulation, total immobilization time, or complication rates between a group who underwent treatment with percutaneous pinning and a group that underwent casting for forearm fracture after six months [21]. Regardless of the controversy surrounding recommendations of recent scientific literature, the standard of care for pediatric diaphyseal forearm fractures remains non-operative treatment with closed reduction and casting [22,23].

In totality, these findings indicate that the recent trends toward increased incidence of operative treatment for pediatric forearm fractures, as observed in the present study, may not align directly with the guidelines and recommendations found in both clinical standards and existing literature, particularly when considering the increased cost of treatment for operative management. The increase in operative treatments for pediatric forearm fractures over the past decade, as revealed by this study, underscores a critical misalignment between clinical practice and the supporting literature/standard of care, which does not conclusively favor surgery over non-operative care. This research supports the need for re-evaluating current treatment strategies in consideration of both the unsubstantiated clinical trends and the significant financial implications associated with the rising preference for operative management.

This study has several limitations. First, it is not possible to extract more detailed data regarding classifications of injury, including severity and mechanism, and short- and long-term functional outcomes of specific treatments. Importantly, this prevents researchers from gaining information about why a certain treatment modality was selected. Regardless, the scope of this study is to provide a broad overview of treatment trends and still offers the field benefit even if the indication for treatment type is unable to be reported. Additionally, the CPT codes for surgical fixation of this injury do not delineate between the different surgical procedures that the physician may use in the reduction of the fracture, making it impossible to further analyze the potential differences in efficacy between different procedures. Further, as with any retrospective database study, our study relies on the precise coding of CPT or ICD code information and therefore runs the risk of inaccurate diagnostic descriptions due to inaccurate documentation, misdiagnosis, or duplicate billing. Notably, CPT and ICD codes are for billing purposes rather than clinical purposes, which puts the data at risk of missing key components. Last, while the present study did not stratify by age and included patients up to the age of 21, this broad inclusion criteria allows us to fully encompass the entire pediatric spectrum. This comprehensive approach ensures that no aspect of the trend in forearm fracture treatment is overlooked, providing a complete picture of management practices from childhood up until young adulthood.

Over the past decade, there has been a significant rise in operatively managed pediatric forearm fractures, which has led to a corresponding increase in treatment costs. It is essential that this trend be supported by robust data, particularly given the prevalence of these injuries and the financial impact on patients and their families. While some evidence suggests the effectiveness of surgical intervention, the overall evidence is not definitive. Further research targeting specific age ranges is necessary to establish clearer guidelines for when surgical intervention should be indicated. With more precise studies, unnecessary surgeries could be avoided, leading to reduced healthcare costs and more tailored treatment protocols. 

## Conclusions

This retrospective cohort study examines trends in the management of patients aged 21 or younger with radial and ulnar shaft fractures using the TriNetX global research database. We observed an increase in the surgical management of these fractures from 2014 to 2023 despite stable reimbursement rates for both operative and non-operative treatments. This trend suggests a shift towards more aggressive intervention, although the clinical efficacy across all pediatric age groups remains to be definitively proven. Future research is necessary to refine treatment protocols, ensuring that surgical interventions are optimally used to enhance outcomes and minimize unnecessary healthcare expenditures.
